# Functional GnRH receptor signaling regulates striatal cholinergic neurons in neonatal but not in adult mice

**DOI:** 10.3389/fendo.2024.1353151

**Published:** 2024-01-29

**Authors:** Imre Farkas, Katalin Skrapits, Miklós Sárvári, Balázs Göcz, Szabolcs Takács, Éva Rumpler, Erik Hrabovszky

**Affiliations:** Laboratory of Reproductive Neurobiology, Institute of Experimental Medicine (Hungarian Research Network), Budapest, Hungary

**Keywords:** caudate-putamen, cholinergic interneuron, gonadotropin-releasing hormone, neonatal mice, spiny projection neuron, striatum 2

## Abstract

Reproduction in mammals is controlled by hypothalamic gonadotropin-releasing hormone (GnRH) neurons. Recent studies from our laboratory established that the basal ganglia of the human brain contain additional large populations of GnRH synthesizing neurons which are absent in adult mice. Such extrahypothalamic GnRH neurons mostly occur in the putamen where they correspond to subsets of the striatal cholinergic interneurons (ChINs) and express GnRHR autoreceptors. In an effort to establish a mouse model for functional studies of striatal GnRH/GnRHR signaling, we carried out electrophysiological experiments on acute brain slices from male transgenic mice. Using PN4-7 neonatal mice, half of striatal ChINs responded with transient hyperpolarization and decreased firing rate to 1.2 µM GnRH, whereas medium spiny projection neurons remained unaffected. GnRH acted on its specific receptor because no response was observed in the presence of the GnRHR antagonist Antide. Addition of the membrane-impermeable G protein-coupled receptor inhibitor GDP-β-S to the internal electrode solution eliminated the effect of GnRH. Further, GnRH was able to inhibit ChINs in presence of tetrodotoxin which blocked action potential mediated events. Collectively, these data indicated that the receptor underlying the effects of GnRH in neonatal mice is localized within ChINs. GnRH responsiveness of ChINs was transient and entirely disappeared in adult mice. These results raise the possibility to use neonatal transgenic mice as a functional model to investigate the role of GnRH/GnRHR signaling discovered earlier in adult human ChINs.

## Introduction

Fertility in mammals is controlled by preoptic/hypothalamic gonadotropin-releasing hormone (GnRH) neurons which release GnRH episodically into the hypophysial portal circulation to control hypophysial gonadotropes. In laboratory rats and mice, the majority of GnRH neurons are located in the preoptic area where they subserve reproductive functions (1). In contrast, the primate brain also contains additional GnRH neurons expressing *GNRH1* mRNA or GnRH immunoreactivity in extrahypothalamic regions unrelated to reproduction, including several basal ganglia and the basal forebrain (2–7).

In recent studies from our laboratory (7), we established that the number of such extrahypothalamic GnRH neurons in the human brain can be as high as ~150,000-200,000 which exceeds considerably the number of hypothalamic GnRH neurons. Such neurons mostly occur in the putamen, synthesize the *bona fide* GnRH decapeptide, and overlap with a subpopulation of locally projecting cholinergic interneurons (ChINs) (7). Using bulk-sequencing of laser capture-microdissected ChINs identified by their large profile area, we have also established that these neurons express GnRHRs, whereas the major target cells of ChINs, spiny projection neurons (SPNs), are devoid of *GNRHR* mRNA (7). Collectively, these data indicated that GnRH is involved in local autocrine/paracrine signaling mechanisms in the human striatum. While presence of the *bona fide* GnRH peptide has not been reported in the striatum of adult mice, transgenic strains generated using various promoter fragments expressed the reporter gene and wild-type mice expressed GnRH immunoreactivity at extrahypothalamic sites during embryonic and early postnatal life (8, 9).

The absence of GnRH expression in the striatum of laboratory rodents hinders functional studies of striatal GnRH/GnRHR signaling. Therefore, the role of the massive *GNRH1* expression in human ChINs remains elusive. In search for an animal model, we carried out a series of slice electrophysiology experiments on ChINs of neonatal and adult transgenic mice.

## Materials and methods

### Animals

Experiments involving genetically modified male mice were carried out in accordance with the Institutional Ethical Codex, Hungarian Act of Animal Care and Experimentation (1998, XXVIII, section 243/1998) and the European Union guidelines (directive 2010/63/EU), and with the approval of the Institutional Animal Care and Use Committee of the Institute of Experimental Medicine. The animals were housed under standard conditions (lights on between 06.00 and 18.00 h, temperature 22 ± 1°C, chow and water *ad libitum*) and all measures were taken to minimize potential stress or suffering during sacrifice and to reduce the number of animals to be used. i) ChAT-Cre/zsGreen mice were generated by crossing ChAT-IRES-Cre knock-in mice (Jackson Laboratory, Bar Harbor, ME; RRID: IMSR_JAX:006410) with the Ai6(RCL-ZsGreen) indicator strain (The Jackson Laboratory, JAX No. 007906) and as used, were heterozygous for both the Cre and the indicator gene alleles. Other previously characterized transgenic models used for slice electrophysiology included ii) GnRH-GFP transgenic mice which selectively express enhanced green fluorescent protein in GnRH neurons (10) and iii) gad65-GFP mice showing selective GFP expression in GABAergic neurons (11).

### Immunofluorescent detection of choline acetyltransferase in GnRH-GFP neurons

#### Perfusion-fixation and section preparation

Male GnRH-GFP transgenic mice from postnatal week 1 (PNW1), PNW2 and PNW4 (N=3/age group) were anesthetized between 0900 and 1100 h with a cocktail of ketamine (25 mg/kg), xylavet (5 mg/kg) and pipolphen (2.5 mg/kg) in saline, and then, perfused transcardially with 4% freshly prepared paraformaldehyde in 0.1 M phosphate buffered saline (PBS; pH 7.4). The brains were removed, infiltrated with 20% sucrose overnight and snap-frozen on powdered dry ice. 25-μm-thick coronal sections were collected from the striatum with a freezing microtome and stored at -20°C in antifreeze solution. These levels correspond to Atlas plates 20-30 of the adult mouse brain according to Paxinos (Bregma levels 1.34-0.14 mm) (12).

#### Perfusion-fixation and section preparation for anatomical studies

Floating sections were pretreated with 0.5% H_2_O_2_ and 0.2% Triton X-100. Cholinergic neurons were detected with the AB144P goat ChAT antiserum (1:2,000; 24h; Merck) (7, 13), followed by biotinylated secondary antibodies (donkey anti-goat IgG; 1:500; 1h; Jackson ImmunoResearch), ABC Elite reagent (Vector; 1:1,000; 1h) and Cy3-tyramide (diluted 1:1,000 with 0.05M Tris-HCl buffer, pH 7.6, containing 0.005% H_2_O_2_; 30 min) (14). Dual-labeled sections were mounted on glass slides, coverslipped with Mowiol and analyzed with confocal microscopy.

#### Confocal microscopy

Fluorescent signals were studied with a Zeiss LSM780 confocal microscope. High-resolution images were captured using a 20×/0.8 NA objective, a 0.6–1× optical zoom and the Zen software (CarlZeiss). Different fluorochromes were detected with laser lines 488 nm for FITC and 561 nm for Cy3. Emission filters were 493–556 nm for FITC and 570–624 nm for Cy3. To prevent emission crosstalk between the fluorophores, the red and the green channels were recorded separately using the “smart setup” function. To illustrate the results, confocal Z-stacks were merged using maximum intensity Z-projection (ImageJ). The final figures were adjusted in Adobe Photoshop using the magenta-green color combination and saved as TIF files.

#### Slice electrophysiology

##### Brain slice preparation for electrophysiological recordings

Brain slices of the different transgenic mice were prepared as described elsewhere (15) and used to record PNW1 (postnatal day 4-7, PN4-7) GnRH-GFP (n=13), PN4-7 ChAT-Cre/zsGreen (n=41), adult (PN80-100) ChAT-Cre/zsGreen (n=10), and PN4-7 GAD65-GFP (n=16) neurons. The mice were decapitated in deep inhalation anesthesia with Isoflurane. The brains were removed and immersed in ice-cold low-Na cutting solution bubbled with carbogen (mixture of 95% O_2_ and 5% CO_2_). The cutting solution contained the following (in mM): saccharose 205, KCl 2.5, NaHCO_3_ 26, MgCl_2_ 5, NaH_2_PO_4_ 1.25, CaCl_2_ 1, glucose 10. CPU blocks were dissected, and 200-μm-thick coronal slices were prepared with a VT-1000S vibratome (Leica Biosystems) in ice-cold oxygenated low-Na cutting solution. The slices were transferred into carbogenated artificial cerebrospinal fluid (aCSF) containing in mM: NaCl 130, KCl 3.5, NaHCO_3_ 26, MgSO_4_ 1.2, NaH_2_PO_4_ 1.25, CaCl_2_ 2.5, glucose 10, and allowed to equilibrate for 1 h while temperature was allowed to drop slowly from 33°C to room temperature.

Recordings were carried out in carbogenated aCSF at 33°C using Axopatch-200B patch-clamp amplifier, Digidata-1322A data acquisition system, and pCLAMP 10.4 software (Molecular Devices Co., Silicon Valley, CA, USA). The patch electrodes (OD = 1.5 mm, thin wall; WPI, Worcester, MA, USA) were pulled with a Flaming-Brown P-97 puller (Sutter Instrument Co., Novato, CA, USA). Neurons were visualized with a BX51WI IR-DIC microscope (Olympus Co., Tokyo, Japan). GnRH-GFP, ChAT-Cre/zsGreen and GAD65-GFP neurons showing green fluorescence were identified by brief illumination at 470 nm using an epifluorescent filter set.

Whole-cell patch-clamp measurements started with a control recording (2 min). Then, a single bolus of GnRH (final 1.2 µM) was pipetted into the measurement chamber filled with aCSF and the recording continued for a further 13 min. In case of the various extracellular blockers, pretreatment of slices with the GnRHR antagonist Antide (100 nM) or the voltage-gated Na-channel inhibitor tetrodotoxin (TTX, 660 nM) started 10 min before GnRH was added to the aCSF. The above inhibitors were present in the aCSF throughout the recording. In other experiments, GPCRs were blocked in the recorded neurons by adding the membrane-impermeable Guanosine 5′-[β-thio]diphosphate trilithium salt (GDP-β-S, 2 mM) (16–21) to the intracellular pipette solution. To allow the intracellular milieu to reach equilibrium, GnRH was only applied 10 min after achieving the whole-cell patch clamp configuration. Each neuron served as its own control when drug effects were evaluated.

### Whole-cell patch clamp experiments

The action potentials (APs) and resting potentials (V_rest_) were recorded in current-clamp mode. V_rest_ was measured at 0 pA in the presence of 660 nM TTX. APs were triggered with a 15 min-long 10 pA depolarizing current pulse throughout the recording. The intracellular pipette solution contained (in mM): HEPES 10, KCl 140, EGTA 5, CaCl_2_ 0.1, Mg-ATP 4, Na-GTP 0.4 (pH 7.3 with NaOH). The resistance of the patch electrodes was 2–3 MΩ. Spike-mediated transmitter release was blocked in some experiments by adding the voltage-sensitive Na-channel inhibitor TTX (660 nM, Tocris) to the aCSF 10 min before GnRH was added to the aCSF.

### Drugs

Extracellularly used drugs: GnRH decapeptide (1.2 µM, Merck); GnRH-R antagonist Antide (100 nM; Bachem, Bubendorf, Switzerland); voltage-gated Na-channel inhibitor tetrodotoxin (TTX; 660 nM, Tocris).

Intracellularly applied drug: the membrane impermeable G-protein blocker GDP-β-S (2 mM, Merck).

### Statistical analysis

Recordings were stored and analyzed off-line. Event detection was performed using the Clampfit module of the PClamp 10.4 software (Molecular Devices Co., Silicon Valley, CA, USA).

Firing rates were calculated from the number of APs in the given recording time (3 min or 12 min). All experiments were self-controlled and frequencies following treatments were expressed as percentages of the untreated control periods. Mean frequencies and V_rest_s detected during the control and treatment periods were calculated separately for each type of recording and group data were expressed as mean ± standard error of mean (SEM). Two-tailed Student’s *t*-tests were applied for each individual group and the differences were considered significant at *p*< 0.05. For group comparisons, firing rates or V_rest_ values were compared by One-way ANOVA with repeated measurements, followed by Tukey’s *post hoc* test.

## Results

### Transient GnRH-GFP transgene expression in the striatum of neonatal mice offers a potential functional model

**Figure 1:**
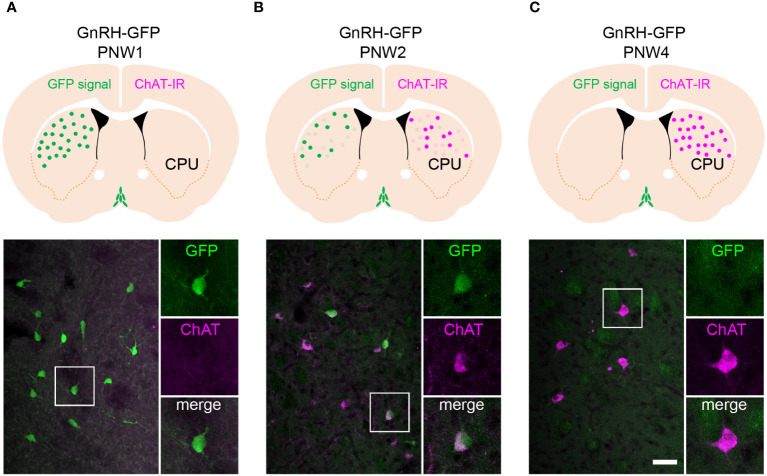
The GnRH-GFP transgene is expressed transiently in striatal cholinergic interneurons of neonatal mice. **(A)** Postnatal week 1 (PNW1) mice exhibit transient green fluorescent protein (GFP) fluorescence in the striatum (caudate-putamen; CPU; green) of GnRH-GFP transgenic mice (10). The cholinergic marker choline acetyltransferase (ChAT; magenta) is not detectable yet with immunohistochemistry at this age. High-power image shows a GnRH-GFP neuron from the framed region. **(B)** PNW2 mice exhibit the GFP signal and also express immunoreactivity to ChAT in the CPU. GnRH-GFP fluorescence occurs selectively within ChAT-immunoreactive cholinergic neurons (high-power inset). **(C)** The ChAT signal becomes much stronger by PNW4. By this time the GnRH-GFP fluorescent signal fades away from cholinergic cells (high-power inset). Scale bar: 50 µm, and 25 µm in insets.

Functional studies of extrahypothalamic GnRH neurons require relevant animal models. While GnRH immunoreactivity or mRNA expression has not been reported in the adult rodent striatum, in a pilot study we noticed that the developing striatum of a GnRH-enhanced green fluorescent protein (GnRH-GFP) transgenic mouse strain (10) transiently expresses green fluorescence. We found that the fluorescent GFP signal was very intense at postnatal week 1 (PNW1) and then, faded gradually to disappear by PNW4 (**Figures 1A–C**). To raise the possibility that these neurons are homologous with the extrahypothalamic cholinergic GnRH neuronal system of the human putamen, we carried out the immunofluorescent detection of the cholinergic marker enzyme, choline acetyltransferase (ChAT) in paraformaldehyde-fixed tissue sections of GnRH-GFP transgenic mice. ChAT immunoreactivity showed an inverse temporal profile in the developing striatum, being nearly undetectable at PNW1 and showing increased expression with development (**Figures 1A–C**). As established with the confocal microscopic analysis of sections from PNW2 mice expressing both GnRH-GFP fluorescence and ChAT immunoreactivity, the transient GnRH-GFP fluorescence characterized selectively a subset of the ChINs (**Figure 1B**). This selective colocalization phenomenon was highly reminiscent of our previous observation in adult humans (7) and raised the challenging possibility that neonatal mice might have a functional value in studies of GnRH effects in the striatum with slice electrophysiology. Therefore, three transgenic mouse strains showing green fluorescence selectively in GnRH-GFP neurons, in cholinergic neurons (ChAT-Cre/zsGreen) and in GABAegic neurons (GAD65-GFP) (11), respectively, were used to study further GnRH effects in the murine striatum with slice electrophysiology.

### GnRH inhibits GnRH-GFP and ChAT-Cre/zsGreen neurons in the striatum of neonatal mice

**Figure 2:**
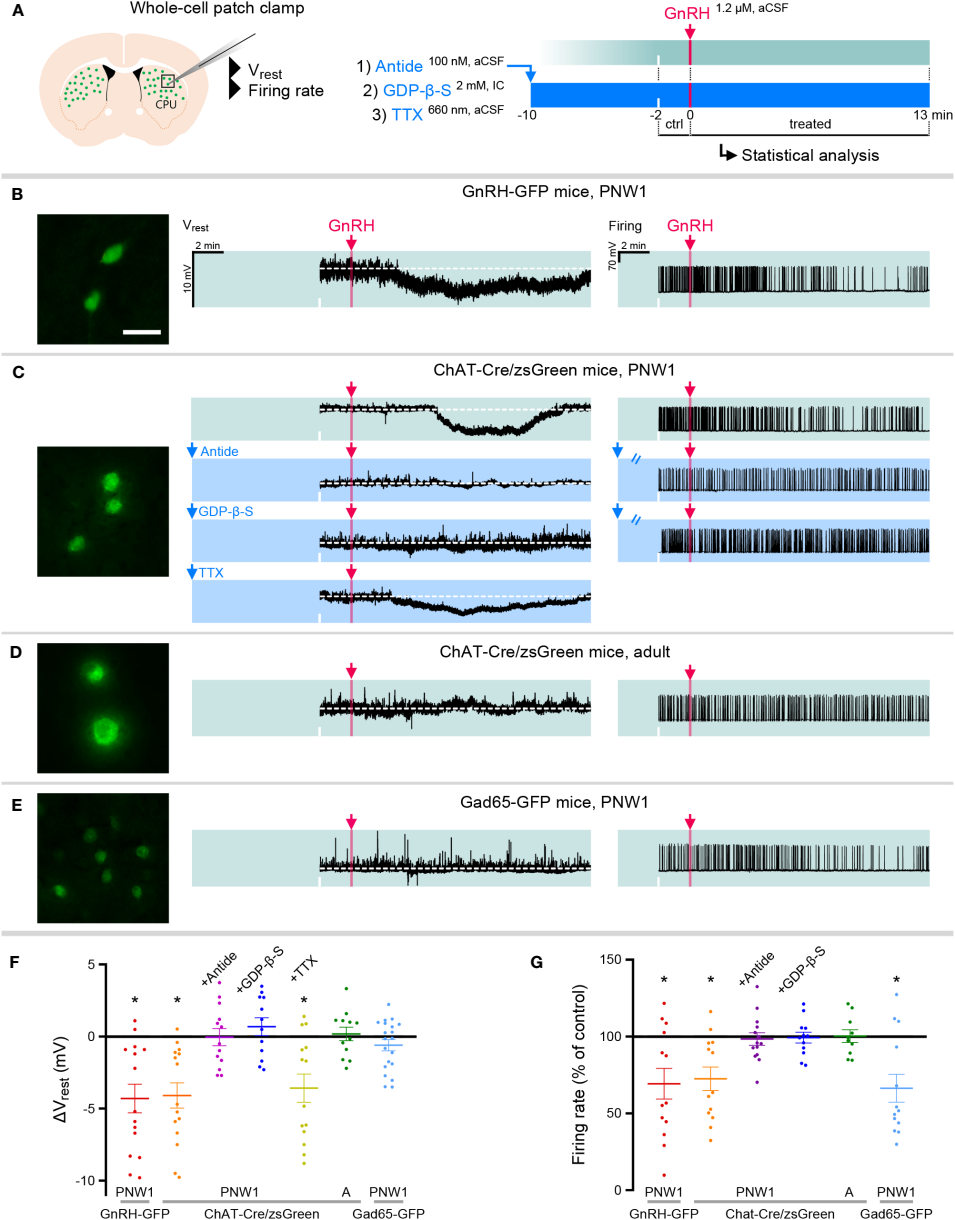
GnRH inhibits cholinergic interneurons in the striatum of neonatal transgenic mice via signaling on GnRHR autoreceptors. **(A)** Neonatal GnRH-GFP transgene expression within cholinergic neurons of the striatum (caudate-putamen; CPU) indicates that newborn transgenic mice may serve as animal model to study GnRH effects with slice electrophysiology. To reveal receptor mechanisms, the selective GnRHR antagonist Antide, the membrane-impermeable G Protein-Coupled Receptor inhibitor GDP-β-S and the action potantial inhibitor tetrodotoxin (TTX) were used in whole-cell patch-clamp experiments. **(B)** Postnatal week 1 (PNW1; postnatal day 4-7) GnRH-GFP neurons responded to GnRH with reduced resting membrane potential (Vrest) and decreased rates in current pulse-induced firing activity. **(C)** The same inhibitory responses could also be elicited from cholinergic interneurons of newborn ChAT-Cre/zsGreen. GnRH acted via its specific receptor GnRHR because inhibitory responses could be prevented with Antide. GnRHR mediating GnRH effects was localized within CPU cholinergic neurons. First, GnRH was unable to inhibit cholinergic neurons if the internal electrode solution contained GDP-β-S. Second, GnRH was still able to hyperpolarize cholinergic neurons in the presence of TTX to eliminate activity-dependent indirect actions (TTX+GnRH). **(D)** In contrast to the newborn mice, adult ChAT-Cre/zsGreen animals did not respond to GnRH with reduced Vrest or firing rate. **(E)** Medium spiny projection neurons which receive input from cholinergic interneurons were studied in neonatal GAD65-GFP transgenic mice. GnRH did not change the Vrest but decreased the firing rate of these neurons, indicating together that the inhibitory response is indirect. **(F, G)** Scatter dot plots summarize the results of measurements in the different treatment groups. *=p<0.05 shows significant change comparing to the lack-of-change values (0 mV in Vrest and 100% in firing rate), obtained by one-sample t-test. ‘A’ refers to scatter dot data from Adult animals in Figures **(F, G)** Scale bar: 25 µm. See also source data for recordings.

In whole-cell patch-clamp experiments on PNW1 (PN4-7) mice (**Figure 2A**), 7 out of 15 striatal GnRH-GFP neurons responded to 1.2 µM GnRH with a transient hyperpolarization (V_rest_ = -51.0 ± 1.11 mV, ΔV_rest_ = -4.3 ± 0.99 mV, **Figure 2B**; p=0.0007). This response started within 2.7 ± 2.1 min and persisted for 8.0 ± 4.5 min following a single bolus of GnRH (final 1.2 µM). To address GnRH effects on neuronal activity, action potentials (APs) were induced by a 10 pA/15-min-long depolarizing current pulse. GnRH transiently decreased the firing rate in 7 out of 13 GnRH-GFP neurons to 69.3 ± 10.01% of the control rate (1.36 ± 0.06 Hz, **Figure 2B**; p=0.0098). GnRH elicited similar inhibitory responses from ChINs of PNW1 ChAT-Cre/zsGreen mice and hyperpolarized 8 out of 15 cholinergic neurons (V_rest_ = -53.6 ± 2.48 mV, ΔV_rest_ = -4.1 ± 0.87 mV, **Figure 2C**; p=0.0004). Furthermore, in 7 out of 13 neurons, GnRH decreased transiently the current pulse-induced firing activity to 72.5 ± 7.61% of the 1.05 ± 0.13 Hz control value (**Figure 2C**; p=0.0098).

### GnRH-dependent inhibition of neonatal cholinergic neurons is mediated by specific GnRH receptors

To identify the receptor involved in the GnRH-induced inhibition of neonatal ChAT-Cre/zsGreen neurons, slices were preincubated with the selective GnRH receptor (GnRHR) antagonist Antide (100 nM) for 10 min prior to GnRH administration (**Figure 2A**). In the presence of Antide, GnRH was unable to affect the resting membrane potential (N=13; **Figure 2C**) and the firing rate (N=14; **Figure 2C**) of ChINs, indicating that GnRH acts on its specific receptor, GnRHR. Based on our RNA-Seq data obtained from human ChINs (7), we hypothesized that the receptor underlying the above effects is present within ChINs as an autoreceptor.

### GnRH acts on GnRHR which is localized to ChINs

GnRHR is a G-protein coupled receptor (GPCR). When the membrane-impermeable GPCR inhibitor GDP-β-S (2 mM, **Figure 2A**) was added to the internal electrode solution, GnRH was unable to alter the V_rest_ (N=12) and the firing rate (N=12) of ChINs (**Figure 2C**). In addition, when the action potential inhibitor tetrodotoxin (TTX; 660 nM, **Figure 2A**) was present in the aCSF to eliminate activity-dependent transsynaptic events, GnRH was still able to hyperpolarize 7 out of 14 ChINs (V_rest_ = -54.1 ± 1.59 mV, ΔV_rest_ = -3.6 ± 0.97 mV, **Figure 2C**; p=0.0029). Together, these observations indicated that GnRHR mediating the effects of exogenous GnRH on ~50% of CPU ChINs is an autoreceptor localized within the responsive cells.

### GnRH does not influence ChINs in adult mice

The GFP phenotype of striatal neurons in GnRH-GFP transgenic mice gradually faded and disappeared by PNW4, raising the possibility that GnRH/GnRHR signaling may also lose significance during development. Indeed, none of the ChAT-Cre/zsGreen neurons studied from adult mice responded to GnRH with an altered V_rest_ (N=12) or firing rate (N=10) (**Figure 2D**).

### GnRH inhibits medium spiny projection neurons of the striatum via indirect actions

The majority of striatal neurons are medium-sized GABAergic spiny projection neurons (SPNs) which receive strong input from ChINs (22). GnRH did not change the resting membrane potential of putative SPNs, identified as medium-sized GAD65-GFP neurons, in neonatal transgenic mice (N=20; **Figure 2E**). This finding was in accordance with the observation that RNA-Seq studies were unable to reveal *GNRHR* expression in the human SPNs (7). When action potentials were studied, GnRH decreased the firing rate of 9 from 13 GAD65-GFP neurons to 66.3 ± 9.07% of the control frequency (0.84 ± 0.16 Hz, **Figure 2E**; p=0.0030), suggesting that GnRH inhibits a subset of SPNs likely via an indirect action. ANOVA revealed significant effects of GnRH on V_rest_ and firing rates in neonatal GnRH-GFP and ChAT-Cre/zsGreen neurons but not in adult ChAT-Cre/zsGreen neurons (**Figures 2F, G**). Application of Antide or GDP-β-S alone did not change the V_rest_ or the firing rate of the control recording periods (for raw data and statistics, see **Supplementary Table 1**).

## Discussion

This is a follow-up study of a recent report from our laboratory in which we detected and characterized 150,000-200,000 GnRH synthesizing neurons in the basal forebrain and in several basal ganglia of the human brain (7). The majority of human extrahypothalamic GnRH neurons were observed in the putamen where they made up a subpopulation (~30%) of ChINs. Using RNA-Seq studies of laser-capture microdissected neurons, we established that, unlike medium spiny projection neurons, ChINs express *GNRHR* autoreceptors, in addition to *GNRH1*. In the absence of an appropriate animal model, the functional significance of extrahypothalamic GnRH signaling in the human brain remains elusive.

To our knowledge, GnRH synthesis by striatal neurons has not been reported in laboratory rodents. Our efforts to detect GnRH immunoreactivity with immunohistochemistry or mRNA expression with RT-PCR and *in situ* hybridization at this site also failed using samples from either neonatal and adult mice, indicating that, if present, *Gnrh* is expressed at very low levels only in the murine striatum. The transcriptome database of ChINs available from adult mice contains neither *Gnrh* nor *Gnrhr* (23), but similar high-throughput studies are currently unavailable to indicate the presence/absence of the authentic GnRH and its receptor in ChINs of neonatal mice. Thus, the possibility remains that developmental GnRH-GFP expression in ChINs of this specific transgenic strain represents an ectopic expression. Accordingly, specific GnRH promoter fragments were shown to play important roles in restricting the non-specific expression of transgene at extrahypothalamic sites during embryonic and early postnatal life (8 , 9) and such important regulatory sequences might be missing from the mouse line we used here.

### Extrahypothalamic GnRH is involved in the regulation of non-reproductive functions

The role of GnRH is not restricted to the regulation of hypophysial gonadotropin secretion. *GNRHR* is expressed in several peripheral organs, including the uterus, the placenta, the ovaries, the testes, the prostate gland and various tumour types (24). Non-reproductive effects on the central nervous system reviewed recently suggest that GnRH-producing neurons play important roles in postnatal brain maturation, odor discrimination and adult cognition (25). Pyramidal neurons of the human hippocampus and cerebral cortex express high levels of *GNRHR* mRNA and GnRH immunoreactivity (26). GnRH is able to increase hippocampal 17β-estradiol levels, it increases the spontaneous firing of pyramidal neurons, causes overexpression of hippocampal *Gnrhr* mRNA and prevents the adverse effects of amyloid β deposition on working memory (27). Recent data from the Ts65Dn mouse model of Down syndrome has raised the intriguing possibility that GnRH therapy might be useful to improve cognitive deficits in patients with Down syndrome (28).

### GnRH/GnRHR signaling in the striatum

Results of our recent studies on *postmortem* human specimens provided evidence for robust species differences between the primate and the rodent brain and revealed 150,000-200,000 GnRH synthesizing neurons in the basal ganglia and basal forebrain of the human brain (7). RNA-Seq studies clarified that ChINs, but not SPNs, of the putamen express *GNRHR*, indicating that GnRH from intrinsic sources binds to and acts on autoreceptors to regulate currently unknown functions of the cortico-striato-thalamocortical neural pathway (7).

The absence of *Gnrh* and *Gnrhr* expression in adult rodent ChINs makes it difficult to study the fuctions of striatal GnRH signaling. Results of our present study on GnRH-GFP transgenic mice raises the possibility that GnRH is expressed transiently in murine ChINs before PW2. While this finding needs to be confirmed using independent methods, failure of our efforts so far to detect *Gnrh* mRNA or GnRH peptide in the striatum of mice from any developmental stage suggests that, if present, the level of GnRH peptide synthesis is much lower in neonatal mice than in the adult human brain. Similarly, transient expression of *Gnrhr* mRNA and GnRHR protein in murine ChINs currently rely only on the present functional observations on PN4-7 mice in which 1.2 µM exogenous GnRH inhibited ChINs via a mechanism that could be antagonized by the selective GnRHR antagonist Antide. It is important to note that genetic identification of *Gnrhr* neurons failed to detect the expression of the receptor in the striatum (29), which calls for more sensitive molecular methodologies to establish the presence of authentic GnRHR in ChINs of neonatal mice.

Our electrophysiological observations on neonatal transgenic mice require careful interpretation. First, the chloride homeostasis in neonatal neurons, which is different in adults, as well as the relatively high 1.2 µM GnRH dose we used, could influence the direction of the electrophysiological response which was inhibitory. In this context, low doses of GnRH was found to inhibit and high doses to excite GnRH neurons in acute brain slices (30). Potential dose dependence of the electrophysiological responses of ChINs has not been addressed in our present study.

Collectively, the electrophysiological observations in this study provide evidence for GnRHR-mediated direct actions of GnRH on ChINs of neonatal mice which disappear in adult animals. Despite obvious model limitations that one needs to keep in mind, the GnRHR-mediated responses in this mouse model may provide insight into some of the roles of *GNRH* and *GNRHR* expression reported recently in ChINs of the adult human putamen.

## Data availability statement

The original contributions presented in the study are included in the article/**Supplementary Material**. Further inquiries can be directed to the corresponding authors.

## Ethics statement

The animal study was approved by Institutional Animal Care and Use Committee of the Institute of Experimental Medicine. The study was conducted in accordance with the local legislation and institutional requirements.

## Author contributions

IF: Conceptualization, Methodology, Supervision, Writing – original draft, Writing – review & editing. KS: Conceptualization, Funding acquisition, Investigation, Methodology, Visualization, Writing – original draft, Writing – review & editing. MS: Conceptualization, Writing – review & editing. BG: Conceptualization, Writing – review & editing. ST: Conceptualization, Writing – review & editing. ÉR: Conceptualization, Writing – review & editing. EH: Conceptualization, Funding acquisition, Supervision, Writing – original draft, Writing – review & editing.
